# Germplasm evaluation and differentiation in *Polygonatum* kingianum via chloroplast genome resources

**DOI:** 10.3389/fpls.2026.1971675

**Published:** 2026-10-15

**Authors:** Rong Liu, Haili Zhao, Jianhong Xie, Liyan Liang, Jing Wang, Xinping Li, Binbin Liao, Baozhong Duan

**Affiliations:** 1 College of Pharmaceutical Science, Dali University, Dali, China; 2 International Joint Laboratory for the Development and Utilization of Traditional Chinese Medicine Resources in Yunnan Province, Dali, China; 3 College of Life Science, Northeast Forestry University, Harbin, China

**Keywords:** chloroplast genome, germplasm differentiation, germplasm evaluation, molecular markers, Polygonatum kingianum

## Abstract

**Introduction:**

*Polygonatum kingianum* (PK) is an important medicinal crop with diverse germplasm that is difficult to distinguish morphologically, particularly at early developmental stages. Misidentification can lead to inappropriate regional cultivation and yield losses when germplasm is introduced into unsuitable environments. Although chloroplast (cp) genomes provide abundant sequence information, variation among PK germplasm groups has not been systematically translated into practical markers for authentication.

**Methods:**

Here, 39 cp genomes representing seven PK germplasm groups and five related *Polygonatum* species were comparatively analyzed to characterize genomic variation and develop PCR-based markers.

**Results:**

Rather than extensive structural divergence, the genomes exhibited localized sequence variation. In an expanded dataset of 118 PK cp genomes, 1,247 single-nucleotide polymorphisms (SNPs) and 263 indels were identified, with higher variant densities in the single-copy regions than in the inverted repeats. Integration of comparative alignments, nucleotide-diversity profiles, and locus-specific differences identified five regions: *ycf2, clpP, rps7-trnV, atpF-atpH*, and *ycf4-cemA* for marker development. Six primer pairs showed 100% amplification and sequencing success in the tested samples and collectively distinguished the tested PK germplasm groups and related *Polygonatum* species. Phylogenomic analyses further resolved PK as a distinct lineage within sect. *Verticillata* and revealed cp differentiation among PK germplasm.

**Discussion:**

These findings show that cp genomic variation provides additional molecular resources for PK germplasm authentication and characterization and may support the evaluation and utilization of medicinal crop genetic resources.

## Highlights

- Localized chloroplast variation enabled development of practical markers for PK germplasm authentication.
- Six primer pairs successfully distinguished tested PK germplasm groups and related *Polygonatum* species.
- Phylogenomics revealed chloroplast differentiation and supported broader evaluation of PK germplasm resources.

## 1 Introduction

*Polygonatum kingianum* Collett & Hemsl. (PK), one of the official source plants of “Polygonati Rhizoma” listed in the Pharmacopoeia of the People’s Republic of China, is an important medicinal plant and economically valuable crop widely distributed in southwestern China (Duan et al., 2017; Commission, 2020). Its broad distribution across diverse environments is associated with substantial germplasm variation, providing valuable genetic resources for improvement and sustainable production (Shu et al., 2026). However, genetic characterization of PK germplasm remains limited, hindering its effective conservation, breeding, and utilization (Liu and Shi, 2023). Reliable identification is particularly challenging because different PK germplasm groups often exhibit highly similar morphology, especially at the seedling stage, despite differences in biological and agronomic traits. Misclassification may result in unsuitable cultivation practices; for example, waterlogging-sensitive germplasm grown in high-rainfall areas may suffer severe yield losses (Zhao et al., 2018; Yang et al., 2023). Thus, reliable germplasm authentication and genetic characterization are important for PK management and utilization.

Complete chloroplast (cp) genomes have shown potential for identifying PK germplasm. However, their direct application in routine authentication requires high-throughput sequencing and extensive bioinformatic analysis, limiting their practicality for rapid identification (Guo et al., 2020). Instead, complete cp genomes can be used to identify informative short regions for PCR-based marker development. Several conventional loci, including *rbcL*, *matK*, *psbA–trnH*, *trnK*, *trnC–petN*, *atpB–rbcL*, and *rps16*, have been used to identify PK and related *Polygonatum* taxa; however, their discriminatory power remains limited among closely related germplasm (Long et al., 2022; Gao et al., 2023). Unlike conventional barcodes, which examine one or a few predefined regions (Patel et al., 2025), nearly complete cp genomes allow polymorphisms distributed across the organellar genome to be surveyed (Li et al., 2015). This broader survey can identify SNPs, InDels, and variable intergenic regions beyond commonly used barcode loci (Li et al., 2023), thereby increasing the likelihood of detecting informative differences among closely related PK germplasm groups. Therefore, comparative analysis of complete chloroplast genomes, followed by the selection of informative short regions, provides a rationale for developing candidate PCR-based markers for germplasm discrimination. Although previous cp genomic studies have identified variable regions in PK and related *Polygonatum* species, systematic screening of genome-wide chloroplast variation across multiple PK germplasm accessions, as well as experimental validation of cp-derived PCR markers, remains limited. In addition, the genetic relationships among PK germplasm groups require further characterization. Together, these gaps limit PK germplasm identification and genetic evaluation.

To address these gaps, 39 newly generated cp genomes representing seven PK germplasm groups and five related *Polygonatum* species were sequenced, assembled, and comparatively analyzed to characterize cp variation and identify candidate marker regions. Selected regions were subsequently developed into PCR-based markers and experimentally evaluated in the tested PK germplasm groups and related species. Phylogenomic and divergence-time analyses were also conducted to examine cp genetic relationships and provide historical context for lineage differentiation among PK germplasm. Together, these analyses connect cp genomic variation to germplasm differentiation and evaluation in PK.

## 2 Materials and methods

### 2.1 Plant materials and DNA extraction

Thirty-nine fresh leaf samples representing seven PK germplasm groups and five related *Polygonatum* species (**Table 1**) were collected from Yunnan, Xizang, and Hunan, China. The PK samples comprised seven germplasm groups (**Figures 1a–g**: *P. kingianum*1–*P. kingianum*7), and the related species included *P. cyrtonema*, *P. hirtellum*, *P. franchetii*, *P. verticillatum*, and *P. cirrhifolium*. These groups represented local germplasm distinguished by geographical origin and morphological characteristics rather than formally established cultivars. Voucher specimens were identified by Professor Baozhong Duan and deposited in the herbarium of Dali University. Approximately 1.0 g of fresh leaves per sample was collected, immediately frozen in liquid nitrogen, and stored for subsequent DNA extraction. Genomic DNA was extracted using a Plant Genomic DNA kit (Tiangen, Beijing, China) according to the manufacturer’s guidelines. The DNA concentration was measured using a high-sensitivity Qubit 4.0 fluorometer (Life Technologies, Inc.).

**Table 1:** Summary of sample information and cp genome structure.

NO.	Species	Source	Voucher specimen	Total length (bp)	GC content (%)	AT content (%)	LSC length (bp)	SSC length (bp)	IR length (bp)	Gene number	Protein-coding gene number	rRNA gene number	tRNA gene number	GenBank accession
1	*P. kingianum* 1	Yunnan, Yuxi	YN2022HJ435	155824	37.7	62.3	84627	18545	26326	130	84	8	38	OR896628
2	*P. kingianum* 1	Yunnan, Yuxi	YN2022HJ436	155827	37.7	62.3	84630	18545	26326	130	84	8	38	OR896629
3	*P. kingianum* 1	Yunnan, Yuxi	YN2022HJ437	155797	37.7	62.3	84615	18530	26326	129	83	8	38	OR896630
4	*P. kingianum* 1	Yunnan, Yuxi	YN2022HJ438	155797	37.7	62.3	84616	18529	26326	129	83	8	38	OR896631
5	*P. kingianum* 1	Yunnan, Yuxi	YN2022HJ439	155825	37.7	62.3	84627	18546	26326	130	84	8	38	OR896632
6	*P. kingianum* 1	Yunnan, Yuxi	YN2023HJ472	155824	37.7	62.3	84627	18545	26326	130	84	8	38	OR896650
7	*P. kingianum* 1	Yunnan, Yuxi	YN2023HJ473	155797	37.7	62.3	84616	18529	26326	129	83	8	38	OR896651
8	*P. kingianum* 1	Yunnan, Yuxi	YN2023HJ474	155827	37.7	62.3	84630	18545	26326	130	84	8	38	OR896652
9	*P. kingianum* 2	Yunnan, Yuxi	YN2022HJ440	155810	37.7	62.3	84628	18530	26326	129	83	8	38	OR896633
10	*P. kingianum* 2	Yunnan, Yuxi	YN2022HJ442	155913	37.6	62.4	84733	18530	26325	129	83	8	38	OR896634
11	*P. kingianum* 2	Yunnan, Yuxi	YN2022HJ443	155809	37.7	62.3	84627	18530	26326	129	83	8	38	OR896635
12	*P. kingianum* 2	Yunnan, Yuxi	YN2023HJ470	155869	37.6	62.4	84672	18547	26325	130	84	8	38	OR896648
13	*P. kingianum* 2	Yunnan, Yuxi	YN2023HJ471	155787	37.7	62.3	84590	18547	26325	130	84	8	38	OR896649
14	*P. kingianum* 3	Yunnan, Dali	YN2022HJ446	155861	37.7	62.3	84633	18532	26348	130	84	8	38	OR896636
15	*P. kingianum* 3	Yunnan, Dali	YN2022HJ447	155862	37.7	62.3	84633	18533	26348	130	84	8	38	OR896637
16	*P. kingianum* 3	Yunnan, Dali	YN2022HJ448	155861	37.7	62.3	84633	18532	26348	130	84	8	38	OR896638
17	*P. kingianum* 3	Yunnan, Dali	YN2022HJ449	155861	37.7	62.3	84633	18532	26348	130	84	8	38	OR896639
18	*P. kingianum* 3	Yunnan, Dali	YN2022HJ450	155861	37.7	62.3	84633	18532	26296	130	84	8	38	OR896640
19	*P. kingianum* 4	Yunnan, Qujing	YN2022HJ451	155729	37.7	62.3	84609	18528	26295	129	83	8	38	OR896641
20	*P. kingianum* 4	Yunnan, Qujing	YN2022HJ452	155719	37.7	62.3	84601	18528	26347	129	83	8	38	OR896642
21	*P. kingianum* 5	Yunnan, Pu’er	YN2023HJ478	155789	37.7	62.3	84569	18526	26347	129	83	8	38	OR896655
22	*P. kingianum* 5	Yunnan, Pu’er	YN2023HJ479	155789	37.7	62.3	84569	18526	26347	129	83	8	38	OR896656
23	*P. kingianum 5*	Yunnan, Yuxi	YN2023HJ476	155775	37.7	62.3	84593	18530	26326	129	83	8	38	OR896653
24	*P. kingianum* 6	Yunnan, Dali	YN2023HJ477	156005	37.6	62.4	84788	18525	26346	130	84	8	38	OR896654
25	*P. kingianum* 6	Yunnan, Yuxi	YN2023HJ482	155911	37.6	62.4	84715	18546	26325	130	84	8	38	OR896659
26	*P. kingianum* 7	Yunnan, Dali	YN2022HJ453	155859	37.7	62.3	84641	18524	26347	130	84	8	38	OR896643
27	*P. kingianum* 7	Yunnan, Dali	YN2022HJ454	155859	37.7	62.3	84641	18524	26347	130	84	8	38	OR896644
28	*P. kingianum* 7	Yunnan, Dali	YN2023HJ529	155688	37.7	62.3	84603	18529	26278	129	83	8	38	OR896663
29	*P. kingianum* 7	Yunnan, Dali	YN2023HJ531	155680	37.7	62.3	84596	18529	26278	132	86	8	38	OR896664
30	*P. kingianum* 7	Yunnan, Dali	YN2023HJ533	155692	37.7	62.3	84606	18530	26278	131	85	8	38	OR896665
31	*P. cyrtonema* 1	Yunnan, Dali	YN2023HJ484	155506	37.7	62.3	84453	18295	26379	128	85	6	37	OR896660
32	*P. cyrtonema* 2	Yunnan, Dali	YN2023HJ485	155492	37.7	62.3	84444	18290	26379	131	85	8	38	OR896661
33	*P. cyrtonema* 3	Yunnan, Dali	YN2023HJ486	155529	37.7	62.3	84464	18307	26379	128	85	6	37	OR896662
34	*P. hirtellum*	Xizang, Changdu	YN2022HJ455	155697	37.7	62.3	84468	18491	26369	130	84	8	38	OR492287
35	*P. franchetii*	Hunan, Zhangjiajie	YN2022HJ456	155909	37.7	62.3	84665	18552	26346	130	84	8	38	OR896645
36	*P. verticillatum*	Yunnan, Dali	YN2022HJ457	155338	37.7	62.3	83944	18564	26415	130	84	8	38	OR896646
37	*P. verticillatum*	Yunnan, Dali	YN2022HJ458	155338	37.7	62.3	83944	18564	26415	130	84	8	38	OR896647
38	*P. cirrhifolium*	Yunnan, Dali	YN2023HJ480	155768	37.7	62.3	84536	18518	26357	130	84	8	38	OR896657
39	*P. cirrhifolium*	Yunnan, Dali	YN2023HJ481	155768	37.7	62.3	84536	18518	26357	130	84	8	38	OR896658

**Figure 1:**
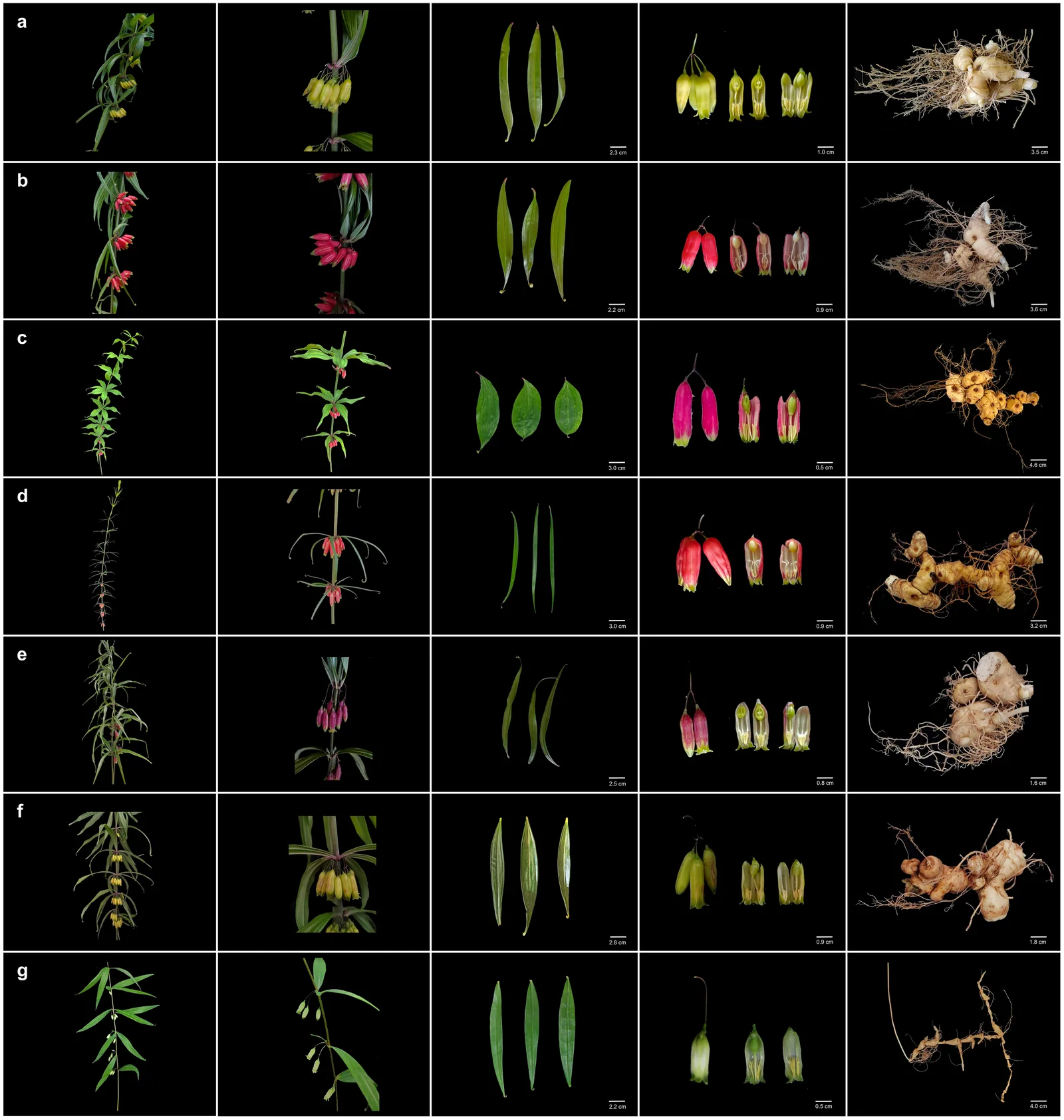
Morphology of *P. kingianum1* **(a)**, *P. kingianum2* **(b)**, *P. kingianum3* **(c)**, *P. kingianum4* **(d)**, *P. kingianum5* **(e)**, *P. kingianum6* **(f)**, *P. kingianum7* **(g)**.

### 2.2 DNA sequencing, assembly, and annotation

For library construction, 30 μL of genomic DNA from each sample at a concentration of at least 100 ng/μL was used. A total of 1 μg of purified DNA was fragmented to construct paired-end libraries with an insert size of approximately 250 bp, which were subsequently sequenced on the Illumina NovaSeq platform (Illumina, San Diego, CA, USA). Raw paired-end reads were quality-filtered to remove adapter sequences and low-quality bases using Toolkit v2.3.3. Chloroplast genomes were assembled using GetOrganelle v1.6.4, with Bowtie2 v2.4.4, SPAdes v3.13.0, and BLAST v2.5.0 as supporting dependencies (Jin et al., 2019). The resulting circular cp genomes were annotated using CpGAVAS2 (http://47.96.249.172:16019/analyzer/annotate) (Shi et al., 2019) and GeSeq (https://chlorobox.mpimp-golm.mpg.de/geseq.html) (Tillich et al., 2017). The annotated genomes were deposited in GenBank under accession numbers OR896628–OR896665 and OR492287 (**Table 1**). Circular chloroplast genome maps were generated using Chloroplot (https://irscope.shinyapps.io/Chloroplot/) (Amiryousefi et al., 2018).

### 2.3 Repeat analysis and comparative analyses

Four types of dispersed repeat sequences, including forward (F), reverse (R), palindromic (P), and complementary (C), were identified using the REPuter tool (https://bibiserv.cebitec.uni-bielefeld.de/reputer/) with a minimum repeat size of 30 bp, a similarity threshold of 90%, and a Hamming distance of three (Kurtz et al., 2001). In addition, simple sequence repeats (SSRs) were analyzed using the MISA software (http://pgrc.ipk-gatersleben.de/misa/) (Beier et al., 2017). The thresholds for mononucleotide, di-nucleotide, trinucleotide, tetra-nucleotide, penta-nucleotide, and hexa-nucleotide motifs were set at ‘10’, ‘5’, ‘4’, ‘3’, ‘3’, and ‘3’, respectively. The genomic coordinates of the identified SSRs were mapped to the corresponding cp genome annotations to determine their genomic locations. Subsequently, SSRs were classified into coding sequences (CDSs), intergenic spacer (IGS) regions, introns, and tRNA/rRNA regions according to the annotated genomic features. Guanine-cytosine (GC) content, genome size, tRNA, and repeat content were assessed using Geneious 9.0.2 software (Kearse et al., 2012). Codon usage bias was evaluated using CodonW v.1.4.2 through six values: effective number of codons (Enc), GC content of synonymous third codon positions (GC3s), codon adaptation index (CAI), frequency of optimal codons (Fop), relative synonymous codon usage (RSCU), and codon bias index (CBI) (Sharp et al., 1986). RSCU values were visualized using standardized bar plots generated with the RSCU-Plot web server (https://pcg-lab.shinyapps.io/RSCU-Plot/), with codons grouped by their corresponding amino acids (Hejazi et al., 2025).

For interspecific chloroplast genome comparisons, three additional *Polygonatum* samples were included as reference taxa: *P. odoratum* (Qingyuan, Guangdong), *P. hunanense* (Zhangjiajie, Hunan), and *P. sibiricum* (Luoyang, Henan). Inverted repeat (IR) contraction and expansion were investigated using IRscope (Amiryousefi et al., 2018). Genome sequences were compared with the mVISTA program in Shuffle LAGAN mode using the annotated information of PK (GenBank MZ029091) as the reference (Katoh and Standley, 2013). Nucleotide variability (Pi) was calculated using DnaSP v.6.12.03 with a 600 bp window and a 200 bp step size (Wang et al., 2022). Pi > 0.01 and Pi > 0.003 were considered high-diversity regions among *Polygonatum* species and among PK accessions, respectively. To further investigate intraspecific genetic variation in PK, 118 PK cp genomes were aligned using MAFFT (https://mafft.cbrc.jp/alignment/software/). Of these, 88 genomes (No. 1–88, **Supplementary Table S1**) were obtained from NCBI, and 30 were newly assembled PK genomes generated in this study (No. 1–30, **Table 1**). Single-nucleotide polymorphisms (SNPs) and insertions/deletions (indels) were identified from the alignment using a Perl script.

### 2.4 Molecular marker development and evaluation

The developed markers were experimentally evaluated using *P. kingianum*, *P. cyrtonema*, *P. hirtellum*, *P. franchetii*, *P. verticillatum*, *P. cirrhifolium, P. odoratum*, *P. hunanense*, and *P. sibiricum.* Candidate coding and intergenic spacer (IGS) regions identified as variable in the comparative analyses were extracted using PhyloSuite v1.2.2 (Zhang et al., 2020). Specific primers targeting selected variable coding and IGS regions were designed using SnapGene v6.2.1 (Insightful Science, available at http://www.snapgene.com). PCR reactions were performed in a total volume of 20 µL, containing 10 µL of 2× Taq Plus PCR Master Mix, 1 µL of each primer, 2 µL of template DNA, and 6 µL of ddH_2_O (Mei5 Biotechnology, Co., Ltd). Amplification was performed using a RePure-A PCR system (Applied Biogener, Hangzhou, China) under the following conditions: initial denaturation at 95 °C for 3 min; 35 cycles of denaturation at 95 °C for 30 s, primer-specific annealing at 55–60 °C (**Table 2**) for 30 s, and 72 °C for 1 min; followed by a final extension at 72 °C for 5 min. PCR products were examined by 1% agarose gel electrophoresis, and successfully amplified products were subjected to bidirectional Sanger sequencing by Sangon Biotech Co., Ltd. (Shanghai, China).

**Table 2:** Primer design by SnapGene.

Name	Fragment	Primer ID	Base sequence (5’-3’)	Length/bp	Tm/°C	GC/%	Identification object
Primer 1	*ycf2*	*ycf2-F*	CGGTCCTGTTTGGTCAAAT	20	55	47	*Polygonatum* species
		*ycf2-R*	TCCCTGGAGAGATAGTTCTCT	22	55	48	
Primer 2	*rps7-trnV*	*rps7-trnV-F*	CAAGAAGAAGGGGTGATCCAT	21	56	48	*Polygonatum* species
		*rps7-trnV-R*	GCCCCATCGAGAAATAGAACT	21	56	48	
Primer 3	*ycf2*	*ycf2-F*	ACCTCGTGTGGGGCTAATAGT	21	60	52	*P. kingianum* germplasm
		*ycf2-R*	GCAGCTCCGTATCAAGGTCAT	22	59	52	
Primer 4	*ycf4-cemA*	*ycf4-cemA-F*	GGGGAACGTATATGGAACAACT	22	57	45	*P. kingianum* germplasm
		*ycf4-cemA-R*	TTCGAAATGTTGCACCGTCT	20	57	45	
Primer 5	*atpF-atpH*	*atpF-atpH-F*	ATCACTTGACTCGCCCTTT	18	56	47	*P. kingianum* germplasm
		*atpF-atpH-R*	CTCGTTGCCATTTGCTTCT	19	55	47	
Primer 6	*clpP*	*clpP-F*	GATATGGAAGGGAGCCAAAACT	22	57	45	*P. kingianum* germplasm
		*clpP-R*	GGCTATTCCTGGTATTACCCAT	22	57	45	

### 2.5 Phylogenetic analyses

A total of 92 chloroplast (cp) genomes were analyzed, including 39 newly generated in this study (**Table 1**) and 53 retrieved from NCBI (No. 66–118 in **Supplementary Table S1**). Three species, *Maianthemum bifolium* (ON872719), *Heteropolygonatum alternicirrhosum* (MZ150832), and *Heteropolygonatum ogisui* (MZ150833), were selected as outgroup taxa. The complete cp genome sequences and the 75 shared coding sequences (CDSs) were treated as two datasets and aligned separately using MAFFT (https://mafft.cbrc.jp/alignment/server/), followed by manual verification.

Phylogenetic trees were inferred using maximum likelihood (ML) and Bayesian inference (BI). ML analyses were performed using IQ-TREE v1.6.12 (http://www.iqtree.org/) with automatic model selection (-m MFP), 1, 000 ultrafast bootstrap replicates, and 1, 000 SH-aLRT replicates (-bb 1000 -alrt 1000 -nt AUTO) (Katoh and Standley, 2013; Nguyen et al., 2015). BI was performed using MrBayes v3.2.6 (Ronquist et al., 2012). The best-fit substitution models for the cp genome (GTR+G+I) and CDS (GTR+G) datasets were determined using MEGA X v10.2.6 (Mao et al., 2023). BI analyses were run for one million generations, with sampling every 1, 000 generations and discarding the first 25% of trees as burn-in (Ronquist et al., 2012; Jiang et al., 2023).

To assess the phylogenetic resolution of the identified hypervariable regions, ML trees were constructed from each region and their concatenated alignment for the same 92 accessions, following the ML procedure described above. The resulting topologies and branch support values were compared with those of the complete cp genome ML tree. The resulting phylogenetic trees were visualized using FigTree v1.4.3 and the Interactive Tree of Life (iTOL; https://itol.embl.de/).

### 2.6 Divergence-time analysis

Divergence times were estimated from the complete cp genome alignment and the corresponding maximum likelihood (ML) tree using RelTime-ML in MEGA 11 (v11.0.13). Branch lengths were estimated under the Tamura–Nei model with uniform rates among sites; variance was estimated analytically, and all sites were retained. Three age ranges reported for *Polygonatum* were assigned to the most recent common ancestors of the corresponding taxon pairs (Xia et al., 2022): *P. verticillatum* and *P. cirrhifolium* (F1, 0.03–2.94 million years ago [Mya]), *P. odoratum* and *P. cyrtonema* (F2, 1.80–9.74 Mya), and *P. kingianum* and *P. prattii* (F3, 11.16–21.58 Mya). The TimeTree Resource (RRID: SCR_021162) was consulted for comparison (Kumar et al., 2017). These ranges were entered as uniform calibration distributions; all other settings were left at their MEGA 11 defaults. However, because the cp genome is a single, predominantly uniparentally inherited locus, the estimates reflect chloroplast lineage divergence rather than necessarily representing species divergence times.

### 2.7 Ancestral character state reconstruction

Phyllotaxy and perianth color were examined to explore evolutionary patterns in *Polygonatum*. Phyllotaxy and perianth-color data were obtained from the cited taxonomic sources (FRPS, 1990). These were categorized into three types: verticillate (A), alternate (B), and opposite (C). The perianth color was categorized into five types: red (A), yellow (B), purple (C), white (D), and green (E). When multiple perianth colors were reported for a species, the predominant color was used for species-level character coding. For example, *P. cirrhifolium* was coded as white because white-flowered individuals were predominant, although red-flowered individuals were also observed. The MultiState Reconstruction in Bayes Traits (MRBT) method was utilized with RASP software to determine the ancestral character state (Yu et al., 2015).

## 3 Results

### 3.1 Genome features and codon usage

After quality filtering and genome assembly, complete cp genomes were obtained from all 39 samples. The genomes ranged from 155, 338 to 156, 005 bp and exhibited the typical quadripartite structure, including a large single-copy region (LSC, 83, 944 - 84, 788 bp), a small single-copy region (SSC, 18, 290 - 18, 564 bp), and two inverted repeat regions (IRs, 26, 278 - 26, 415 bp) (**Figure 2**). The overall GC content was 37.7%. The cp genomes encoded 129–133 genes, including 83–87 protein-coding genes, 38 tRNA genes, and 8 rRNA genes. Functionally, these genes were categorized into three major groups: photosynthesis-related genes, replication-related genes, and other genes (**Supplementary Table S2**). Among these genes, 19 genes were duplicated in the IR regions, and 19 genes contained introns; *clpP* and *ycf3* contained two introns each, whereas the remaining 17 contained one intron. Overall, the examined cp genomes showed highly similar genome structures and gene compositions.

**Figure 2:**
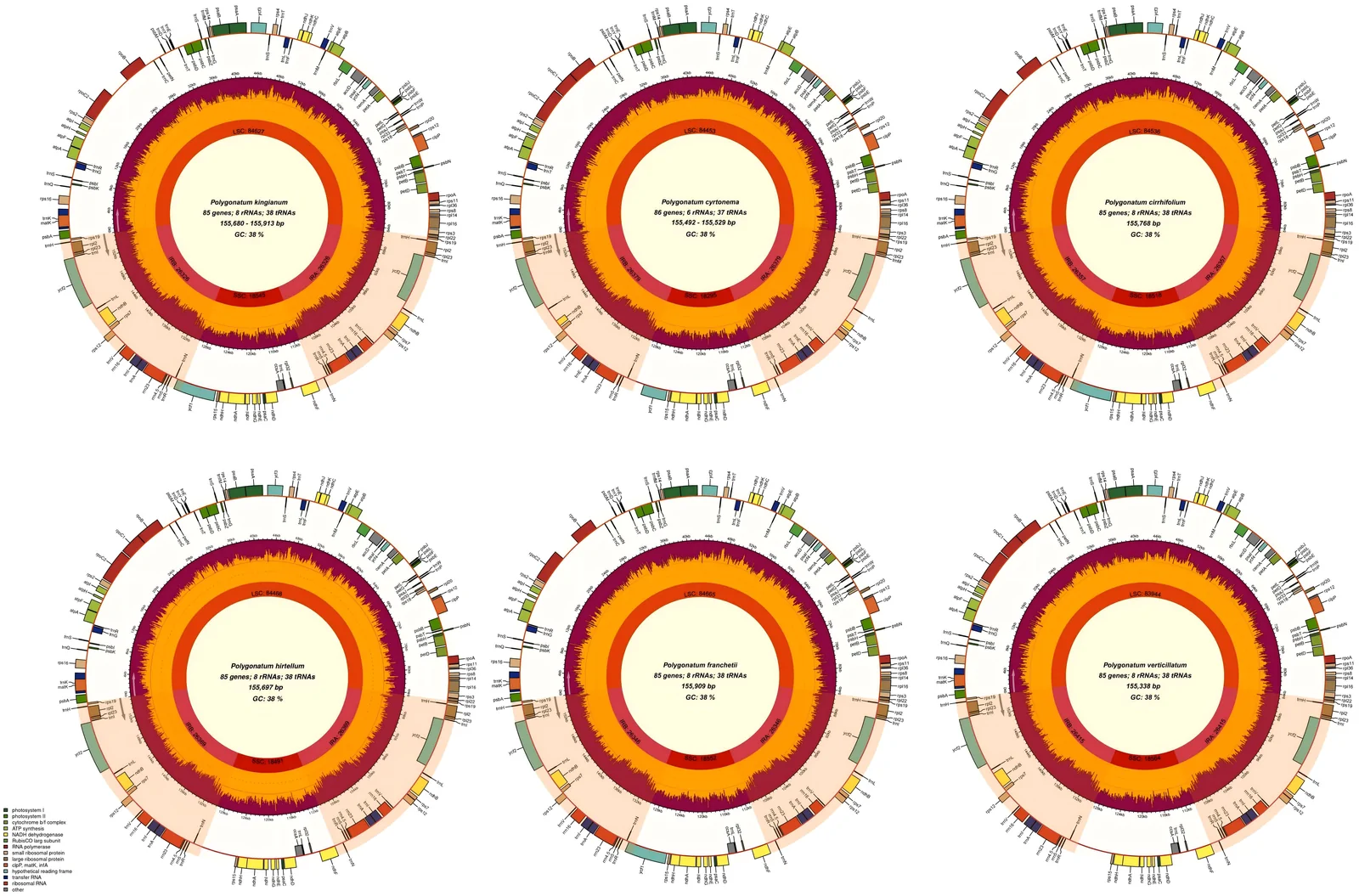
Cp genome map of *Polygonatum* species.

Codon usage was also examined across the cp genomes. A total of 64 codons were identified, including 61 sense codons encoding 21 amino acids and three stop codons. Leucine was the most abundant amino acid (1, 826 - 2, 174), whereas cysteine was the least abundant (191 - 243). AUU was the most frequently used codon, followed by GAA, whereas UGC was the least frequent. Among the 30 codons with RSCU values > 1, 29 ended in A or U, whereas 28 of the 31 codons with RSCU values ≤ 1 ended in G or C (**Supplementary Figure S1**). GC3s ranged from 25.9% to 26.9%, and the effective number of codons ranged from 48.73 to 49.34. Both the codon adaptation index and frequency of optimal codons were below 0.5 (**Supplementary Table S3**).

Repeat analysis identified 35–57 long repeats and 49–64 simple sequence repeats (SSRs). Long repeats ranged from 30 to 71 bp, with 30–39 bp repeats being the most abundant, whereas those longer than 50 bp were rare. F and P repeats predominated over R repeats, and no C repeats were detected among the six species (**Supplementary Figure S2**). SSRs were classified into mono- (A/T), di- (AG/CT, AT/CG), tri- (AAT/ATT, AGC/CTG), tetra- (AAAT/ATTT, AATC/ATTG, AATG/ATTC), penta- (AAAAT/ATTTT), and hexa- (AATAGT/ACTATT) (**Figure 3a**). A/T-rich mononucleotide SSRs predominated and were mainly distributed in the LSC region, with fewer in the IR and SSC regions (**Figure 3b**). Region-specific analysis further showed that SSRs were distributed among CDS, IGS, intron, and tRNA/rRNA regions. Across the 14 examined chloroplast genomes, 306 SSRs were located in CDS regions, 233 in IGS regions, 86 in introns, and 71 in tRNA/rRNA regions (**Figure 3c**; **Supplementary Table S4**).

**Figure 3:**
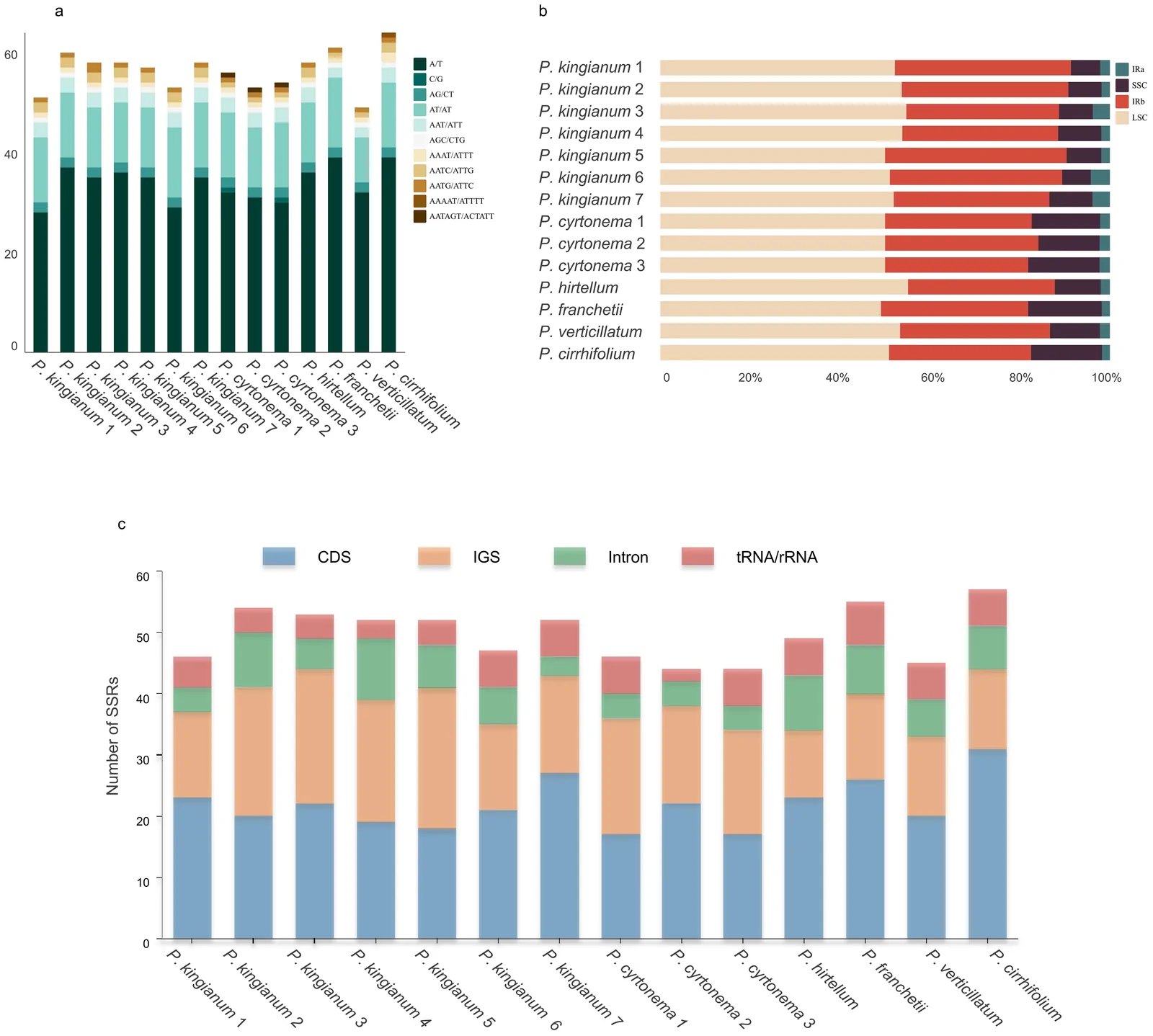
Compares SSR distribution in the cp genomes of 14 *Polygonatum* species. **(a)** Number of types present in 14 cp genomes. **(b)**. Frequency of SSRs in the LSC, IR, and SSC regions; **(c)** Distribution of SSRs among CDS, IGS, intron, and tRNA/rRNA regions.

### 3.2 Chloroplast variation patterns

Comparative sequence analysis revealed localized variation despite the overall conservation of the cp genomes. At the interspecific level, most protein-coding regions were more conserved than non-coding regions, except *ycf1*, *ycf2*, and *clpP*. The greatest variation occurred in the intergenic spacers (IGSs) *rps16* - *trnQ-UUG*, *trnS-GCU* - *trnT-CGU*, *atpA* - *atpH, rpl32 - trnL-UAG*, *trnC - GCA - petN*, *ndhF - rpl32*, *trnT - UGU - trnL - UAA*, *rpoB* - *trnC-GCA*, *petA - psbJ*, and *ccsA - ndhD* (**Supplementary Figure S3**). Within PK germplasm, relatively variable regions included *ycf1*, *ycf2*, *psbK - psbI*, *trnS-GCU* - *trnT-CGU*, *trnT - UGU*-*trnL - UCU*, *petA - psbJ*, *ycf4 - cemA*, and *rps7 - trnV-GAC* (**Supplementary Figure S4**).

Sliding window analysis quantified these sequence differences. Pi values ranged from 0 to 0.02236 among *Polygonatum* species and from 0 to 0.01238 among PK germplasm (**Supplementary Figure S5**). Regions exceeding the defined Pi thresholds were located in the single-copy regions and were predominantly non-coding. At both the interspecific and PK germplasm levels, *ycf1* was the only protein-coding gene exceeding the corresponding threshold.

IR-boundary comparison separately revealed structural differences among the examined cp genomes (**Supplementary Figure S6**). The *rpl2*, *trnH*, and *trnN* genes are entirely located within the IR regions across all *Polygonatum* species, whereas the *rps3*, *rpl22*, and *psbA* genes are located in the LSC region. Additionally, the rps19 gene was fully contained within IRb in all species except *P. sibiricum*, where it occurred at the IRb/LSC boundary. The *ndhF* gene crossed the IRb/SSC boundary in all species and extended 22–34 bp into IRb. The *ycf1* gene crossed the IRb/SSC boundary in *P. kingianum3*, *P. kingianum7*, and *P. odoratum* by 1–13 bp. Its position at the IRa/SSC boundary also varied among the examined taxa, with lengths ranging from 4, 454 to 4, 565 bp.

### 3.3 Marker development and authentication

For marker development, nucleotide-diversity patterns and sequence variants were considered together with the comparative-alignment results. Among *Polygonatum* species, regions with Pi > 0.01 included *matK-trnK*, *trnC-petN*, *trnT-trnL*, *rpoB-trnC*, *petA-psbJ*, *ndhF-rpl32*, *rpl32-trnL*, *ccsA-ndhD*, and *ycf1*, whereas *ycf1* was the only protein-coding gene above this threshold. Among PK germplasm, regions with Pi > 0.003 included *psbK-psbI*, *rpoB-trnC*, *trnC-petN*, *trnY-trnE*, *rps4-trnT*, *ndhC-trnV*, *petA-psbJ*, *rpl32-trnL*, and *ycf1*, with *ycf1* again the only protein-coding gene above the threshold. A total of 1, 510 sequence variants were identified across the 118 PK cp genomes, comprising 1, 247 SNPs and 263 indels (**Supplementary Table S5**). The average SNP and indel densities were 8.2/kb and 1.7/kb across the cp genome, 10.0/kb and 2.4/kb in the LSC region, 14.6/kb and 1.7/kb in the SSC region, and 2.6/kb and 0.6/kb in the IR region, respectively.

Integration of the comparative alignments, nucleotide-diversity profiles, and locus-specific sequence differences identified five fragments for experimental marker development: *ycf2*, *clpP*, *rps7 - trnV, atpF - atpH*, and *ycf4 - cemA*. Six primer pairs were designed from these regions (**Table 2**). Primers 1 and 2 distinguished all *Polygonatum* species included in this study (**Supplementary Figure S7** and **S8**). Among the PK germplasm groups, sequence alignment of the Primer 3 amplicons revealed a 24 bp difference between *P. kingianum* 7 and the other six groups. In contrast, the Primer 4 amplicon from *P. kingianum* 4 showed sequence variation relative to the other groups (**Figure 4**). In addition, Primers 5 and 6 collectively distinguished all tested PK germplasm groups (**Supplementary Figure S9**).

**Figure 4:**
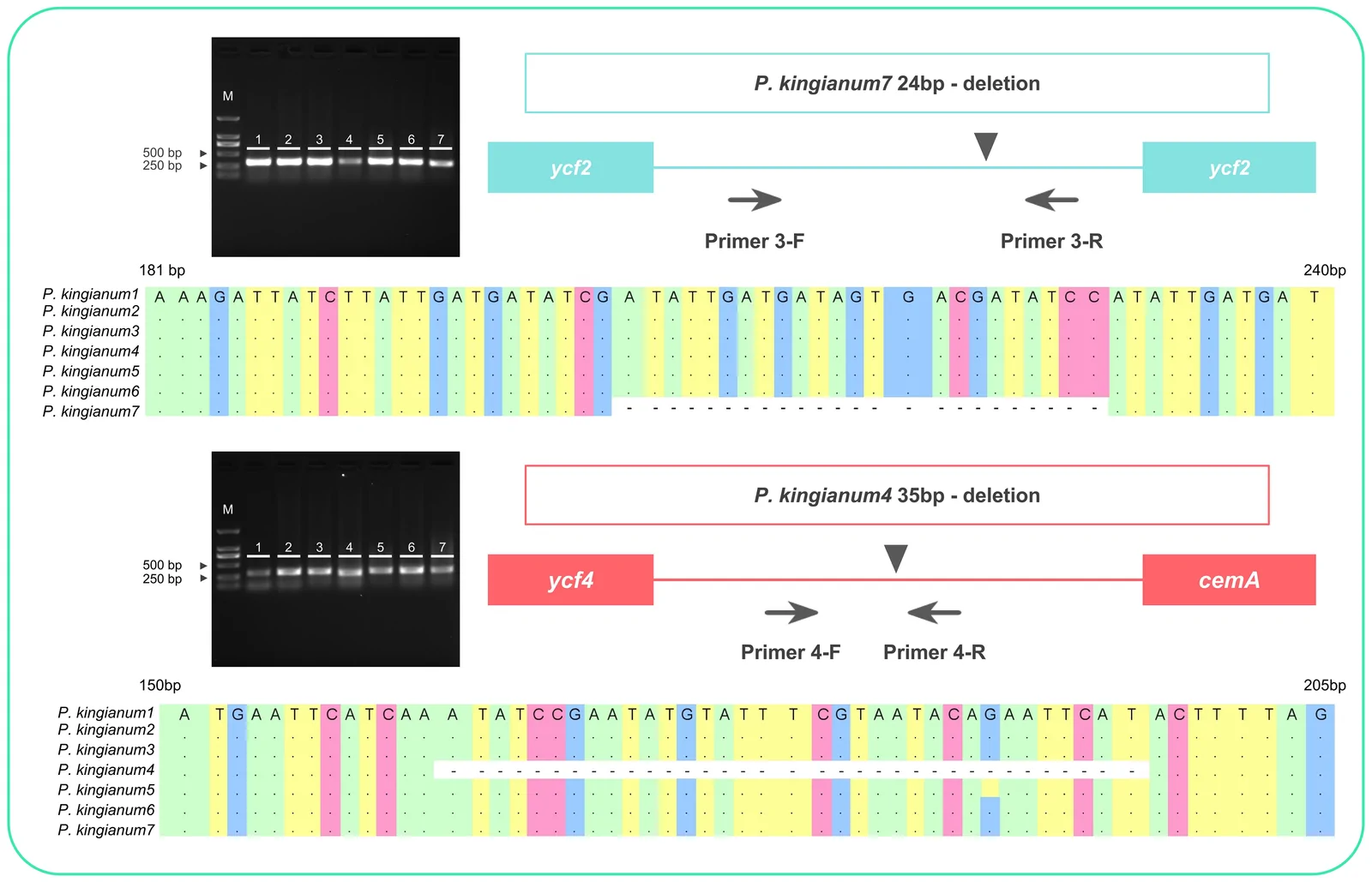
Schematic representation of indel markers for PK germplasm groups. Primer 3 (*ycf2*) and primer 4 (*ycf4-cemA*) were tested. Lane M was the marker of DL 2000; 1. *P. kingianum*1; 2. *P. kingianum*2; 3. *P. kingianum*3; 4. *P. kingianum*4; 5. *P. kingianum*5; 6. *P. kingianum*6; 7. *P. kingianum*7.

Marker performance was evaluated by amplification and sequencing. All six primer pairs showed 100% amplification and sequencing success in the tested samples. The sequencing results were consistent with the corresponding cp genome data and revealed species-specific nucleotide variation. Within the current sample set, the five selected fragments showed discriminatory potential for the tested *Polygonatum* species and PK germplasm groups.

### 3.4 Germplasm diversity and relationships

Cp genomic data were also used to characterize relationships among the examined germplasm. Phylogenetic analyses of the 92 cp genomes, based on cp genome data and shared protein-coding genes, produced nearly identical ML and BI topologies and resolved two major clades (**Figure 5**; **Supplementary Figure S10** and **S11**). Clade I comprised sect. *Verticillata* with 99% support, whereas Clade II contained sect. *Sibirica* and sect. *Polygonatum*, each with 100% support. Within sect. *Polygonatum*, six species were identified, including *P. filipes*, *P. cyrtonema*, *P. humile*, *P. odoratum*, *P. hunanense*, and *P. involucratum*. Among these, *P. filipes* and *P. hunanense* were placed close to *P. cyrtonema*. Sect. *Verticillata* comprised 14 species. Notably, the white-flowered *P. cirrhifolium* was placed close to *P. prattii*, whereas the red-flowered *P. cirrhifolium* was placed close to PK. Additionally, *P. verticillatum* formed a sister group with *P. zanlanscianense*, and *P. franchetii* formed a sister relationship with *P. stenophyllum*. PK formed a distinct branch within Clade I with 100% support, and several PK germplasm groups formed well-supported subclades with 99% or 100% support. *P. kingianum* 1 and *P. kingianum* 2 clustered closely. Both originated from the same geographical region and shared similar traits except for perianth color. *P. kingianum* 6 grouped with *P. kingianum* 1, *P. kingianum* 2, *P. kingianum* 3, and *P. kingianum* 7.

**Figure 5:**
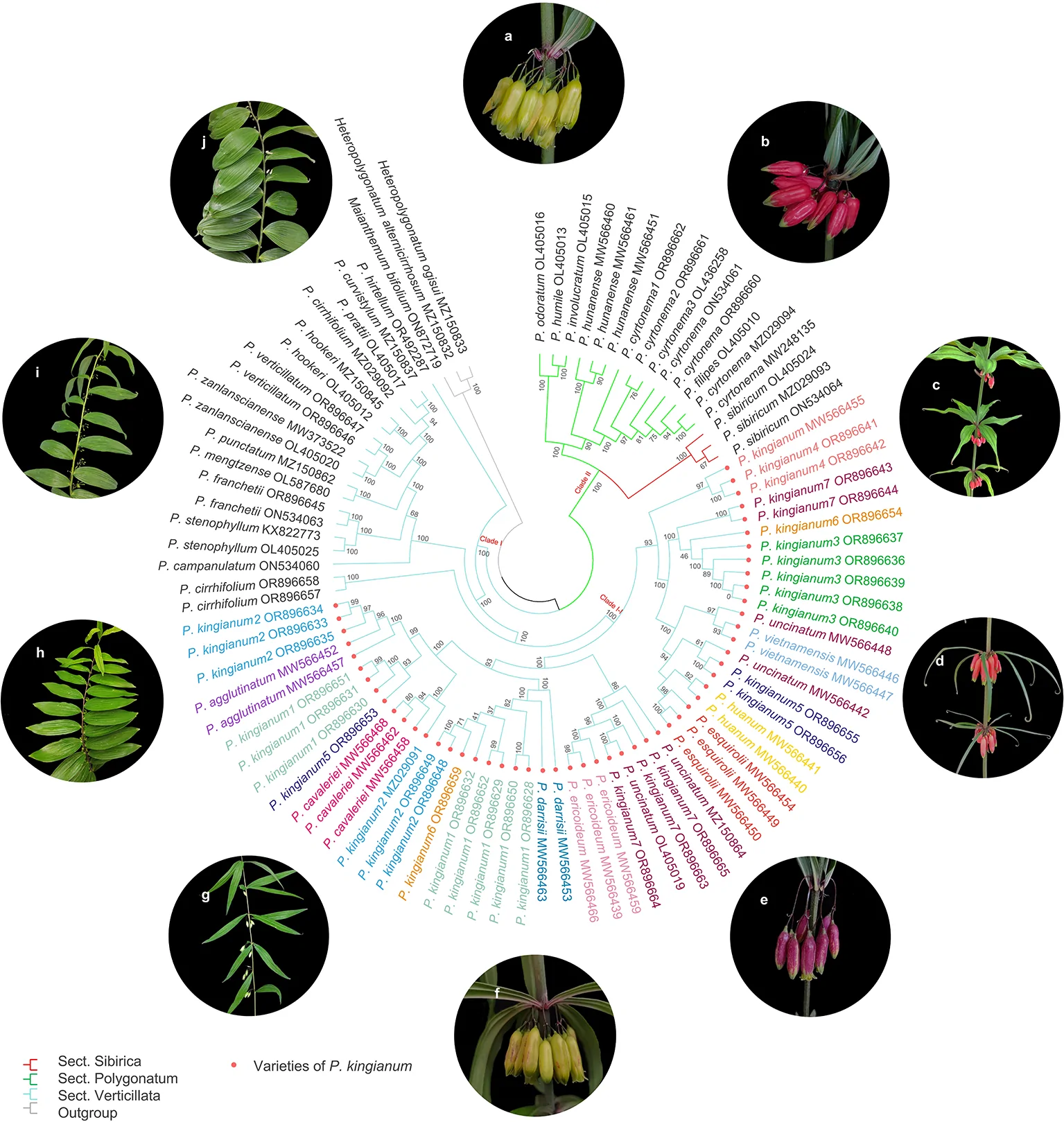
ML phylogenetic tree based on 92 cp genomes. The bootstrap support values are listed at each node. **(A)** *P. kingianum*1; **(B)** *P. kingianum*2; **(C)** *P. kingianum*3; **(D)** *P. kingianum*4; E. *P. kingianum*5; **(F)** *P. kingianum*6; G *P. kingianum*7; H. *P. cyrtonema*1; **(I)** *P. cyrtonema*2; **(J)** *P. cyrtonema*3.

ML trees constructed from the six individual hypervariable regions and their concatenated sequences showed varying degrees of topological congruence with the complete cp genome tree (**Supplementary Figures S12** and **S13**). Notably, the concatenated tree recovered more branches consistent with the complete cp genome tree than any individual-region tree. The three outgroups and three *P. sibiricum* accessions each formed a separate clade in four individual-region trees and the concatenated tree, but not in the *rps7–trnV* or atpF–atpH trees. Among the *P. kingianum* germplasm groups, *P. kingianum* 3 and *P. kingianum* 4 formed distinct clades in both the concatenated and complete cp genome trees, whereas their recovery varied among individual-region trees.

Divergence-time analysis provided a temporal context for the inferred cp relationships. The sampled *Polygonatum* lineages were estimated to have originated approximately 16.20 million years ago (Mya) in the mid-Miocene, followed by diversification of Clade I at 13.83 Mya and Clade II at 11.45 Mya (**Figure 6**). Several *Polygonatum* species differentiated during the Pleistocene. The cp lineages represented by the sampled PK germplasm also showed differentiation during this period, spanning approximately 2.60 - 0.14 Mya.

**Figure 6:**
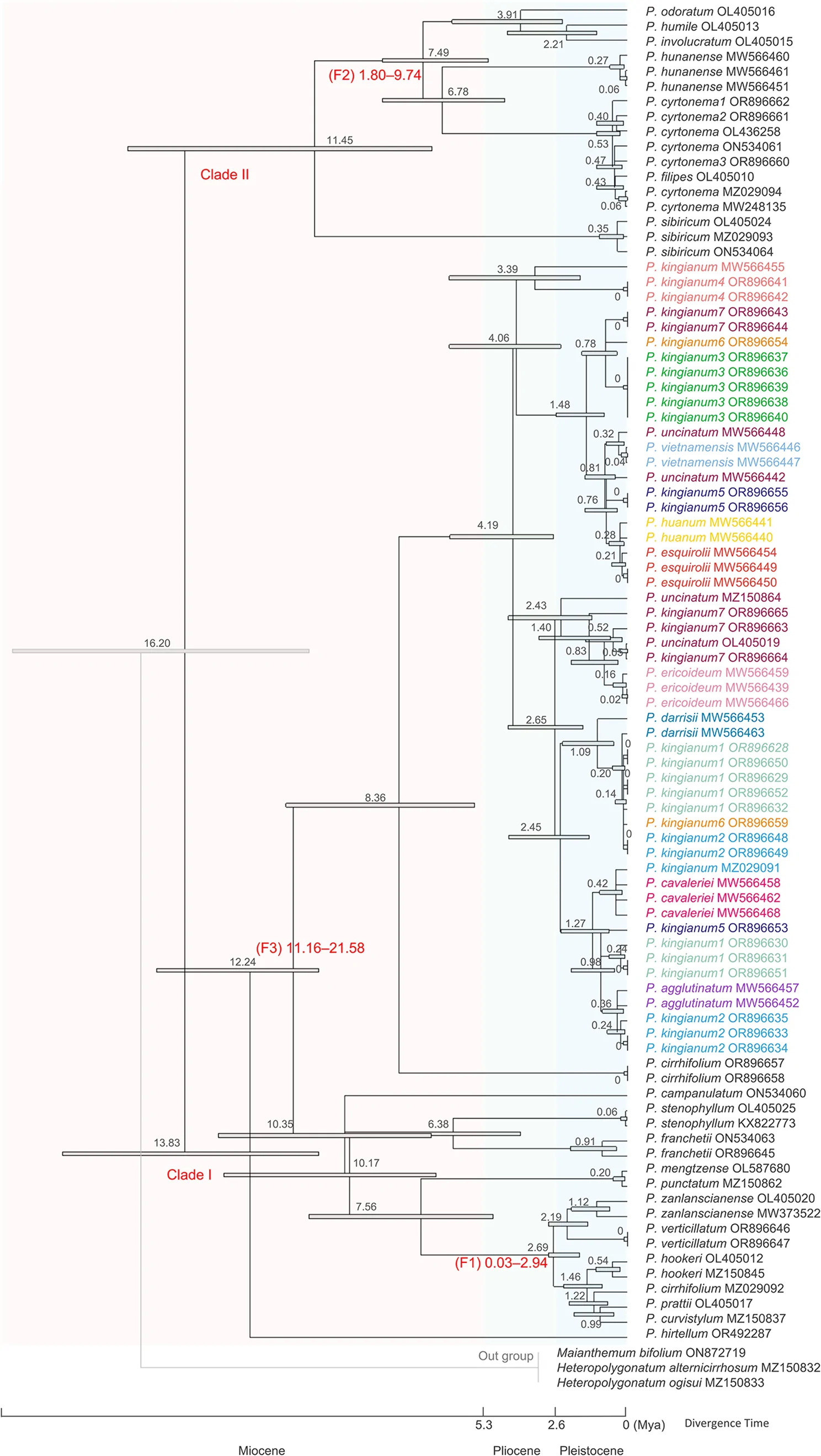
Divergence time estimation based on cp genomes. The node ages are given for each node.

Ancestral-state reconstruction separately examined phyllotaxy and perianth color (**Figure 7**). Verticillate leaves were reconstructed as the ancestral state of *Polygonatum*. Sect. *Verticillata* retained this state, whereas sect. *Sibirica* and sect. *Polygonatum* typically exhibited alternate leaves. Alternate leaves were also present in some typical verticillate species. White flowers were inferred as the likely ancestral state of *Polygonatum*, whereas red flowers were inferred as the ancestral state of PK. Intraspecific variation in perianth color was also observed.

**Figure 7:**
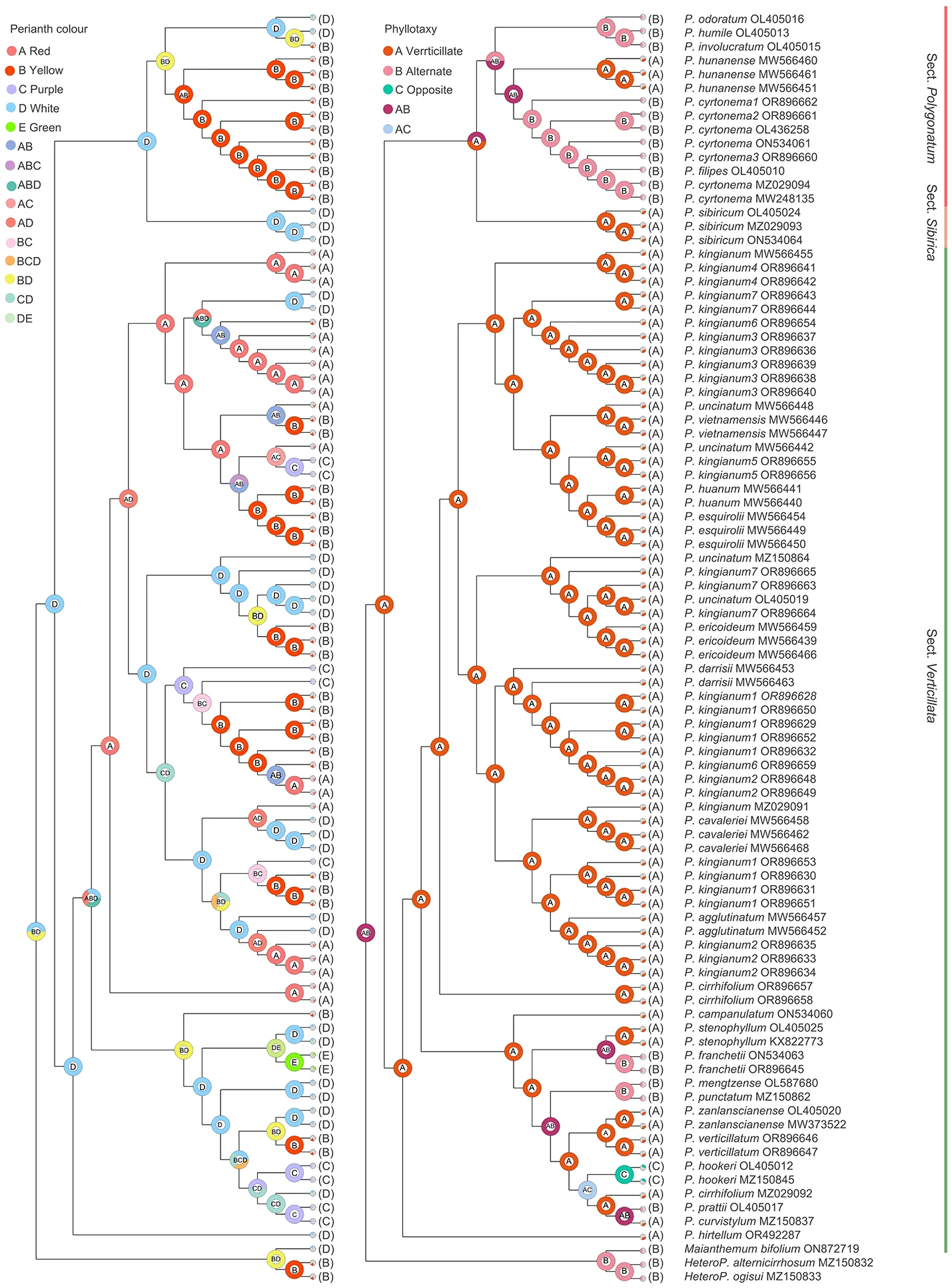
Ancestral state reconstructions of leaf arrangement in genus *Polygonatum*.

## 4 Discussion

### 4.1 Chloroplast variation for PK germplasm authentication

Cp genomic variation among PK germplasm groups provides the genetic basis for developing germplasm-discriminating molecular markers. Although the PK chloroplast genomes were highly conserved in overall structure and gene organization, 1, 247 SNPs and 263 InDels were detected, with most variation occurring in the single-copy regions. Nucleotide diversity and sequence-comparison analyses further identified several variable regions among the germplasm groups. Thus, the chloroplast genomes of PK are characterized not by extensive structural divergence, but by localized sequence variation within an otherwise conserved genomic background. This pattern is relevant to germplasm authentication because such localized differences provide candidate sequence information for distinguishing closely related germplasm (Wang et al., 2022; Shi et al., 2023; Yan et al., 2023).

Similar patterns of localized chloroplast variation have been reported in both *Polygonatum* and other medicinal plants. Previous studies of *Polygonatum* found that cp genomes were generally conserved while several coding and intergenic regions retained relatively high sequence variation (Wang et al., 2022). Cultivar- or genotype-associated cp polymorphisms have also been reported in *Scutellaria baicalensis* and *Dioscorea polystachya*, where SNPs, InDels, or variable regions provided useful information for distinguishing closely related germplasm (Cao et al., 2018; Jiang et al., 2023). These studies support the pattern observed in PK: germplasm-associated variation can be retained in localized chloroplast polymorphisms even when the overall chloroplast genome remains highly conserved.

For PK germplasm authentication, the significance of this variation lies in its potential for marker development rather than in the amount of variation itself. Not every polymorphic region is suitable for practical identification; useful loci must contain stable germplasm-associated differences and support reliable primer design and amplification. The cp variation detected here therefore provides a candidate pool from which informative short sequence regions can be selected for subsequent PCR-marker development (Wang et al., 2024; Hu et al., 2025; Jeong et al., 2025). Because chloroplast genomes represent only one inherited genomic component, however, these differences should not be interpreted as evidence of genome-wide differentiation among PK germplasm groups (Qin et al., 2024).

### 4.2 Molecular markers for PK authentication

The practical value of cp variation lies in whether it can be converted into reliable markers for germplasm authentication (Guo et al., 2022; Xie et al., 2023). In this study, five cp regions (*ycf2*, *clpP, rps7–trnV*, *atpF–atpH*, and *ycf4–cemA*) were selected for marker development, and six primer pairs were designed. All six primer pairs achieved 100% amplification and sequencing success in the tested samples. Sequence alignment of the Primer 3 amplicons identified a 24 bp difference in *P. kingianum* 7, while the Primer 4 amplicon from *P. kingianum* 4 showed sequence variation compared with the other groups. In combination, Primers 5 and 6 differentiated all tested PK germplasm groups. Primers 1 and 2 also distinguished the related *Polygonatum* species included in this study.

Previous studies have demonstrated that conventional DNA barcodes, including ITS, *psbA–trnH*, *rbcL*, *matK*, and related chloroplast loci, are valuable for species-level identification in *Polygonatum* (Long et al., 2022; Wang et al., 2023). For example, *psbA–trnH* aided species identification in a study of 39 medicinal *Polygonatum* samples, although some samples could not be assigned to a species (Jiao et al., 2018). However, such species-level findings do not establish how well conventional barcodes distinguish PK germplasm. In the present study, we explored genome-wide cp variation and selected multiple loci containing polymorphisms among the tested PK germplasm, thereby providing additional sequence resources for its discrimination. Nevertheless, because conventional barcodes were not evaluated in these samples, their discriminatory performance remains unknown. Future side-by-side comparisons of ITS, *matK*, *psbA–trnH*, and cp-derived markers are needed to assess their relative discriminatory power in the same PK germplasm.

These results show that short markers derived from chloroplast genomic variation can provide sequence-based information for distinguishing the PK germplasm tested here. The combined use of multiple markers is particularly important when no single locus can distinguish all germplasm groups (Sun et al., 2024). However, several practical challenges remain before these markers can be used for routine authentication. First, the present study used individual samples, whereas commercial medicinal materials may contain mixtures of different PK germplasms. Therefore, the detection sensitivity and discriminatory capacity of these markers should be evaluated in commercial materials. Second, differences in DNA extraction methods and template quality may affect PCR amplification and sequencing, particularly in processed or degraded materials. These effects should be assessed by comparing different extraction methods and samples of varying DNA quality. Finally, validation in larger independent collections spanning broader geographic and genetic diversity is needed to assess marker robustness and applicability. Future integration with nuclear genomic markers may further improve resolution and provide a more comprehensive framework for PK germplasm authentication.

### 4.3 Chloroplast genomics supports germplasm evaluation

Cp genomic information provides an additional genetic basis for evaluating PK germplasm (Su et al., 2023). In the present study, PK formed a distinct lineage within sect. *Verticillata*, and several germplasm groups formed well-supported subclades with 99–100% support. *P. kingianum*1 and *P. kingianum*2 clustered closely and originated from the same geographical region. Divergence-time analysis further indicated relatively recent differentiation among the cp lineages represented by PK germplasm, mainly during the Pleistocene. Ancestral-state reconstruction also showed changes in phyllotaxy and perianth color during lineage differentiation. These results describe PK germplasm from both genetic-relationship and historical perspectives.

Previous studies have resolved major phylogenetic relationships within *Polygonatum*, identified candidate variable regions, and developed species-specific markers using complete cp genomes and concatenated protein-coding genes (Wang et al., 2016; Xia et al., 2022). In the present study, we evaluated the phylogenetic utility of six selected hypervariable regions across 92 accessions, including PK germplasm. Their concatenation recovered more branches consistent with the complete cp genome tree than the individual regions, although some relationships remained discordant. Thus, these regions retain partial phylogenetic information but cannot fully reproduce the plastome topology. Nevertheless, phylogenetic resolution and marker-based authentication are distinct. The combined use of markers developed from five of these regions enabled authentication of the tested germplasm, highlighting the complementary roles of targeted markers and complete cp genomes in germplasm identification and phylogenetic reconstruction.

The present codon analysis provides another aspect of this genomic information. The examined chloroplast genomes showed a clear preference for A/U-ending codons, with 29 of the 30 codons showing RSCU > 1 ending in A or U. GC3s values were also low (25.9 - 26.9%). Similar A/U-ending preferences are common in chloroplast genomes and are associated with mutational bias and evolutionary constraints (Qin et al., 2024). For PK, these codon-use characteristics complement phylogenetic information by describing a conserved functional feature of the chloroplast genome.

Thus, cp genomes provide more than markers for germplasm authentication. They also provide information on genetic relationships, lineage history, and codon-use characteristics that can support PK germplasm characterization and future breeding research (Xia et al., 2022; Shi et al., 2024). However, the present codon analysis does not demonstrate differences in gene expression or breeding performance. Because the cp genome represents a single, predominantly uniparentally inherited genetic locus, the estimated divergence times reflect chloroplast lineage divergence rather than necessarily corresponding to species divergence times or the origins of the sampled PK germplasm groups. Broader germplasm sampling and nuclear genomic data are still required for a more complete evaluation of PK germplasm.

## 5 Conclusion

In conclusion, comparative analysis of chloroplast genomes revealed localized sequence variation among PK germplasm groups within an otherwise conserved genomic background. Five candidate regions were developed into six primer pairs, which distinguished the tested PK germplasm groups and related *Polygonatum* species within the current sample set. Phylogenomic and divergence-time analyses further revealed chloroplast genetic differentiation among PK germplasm and provided complementary information for evaluating their genetic relationships and lineage history. Together, these findings show that cp genomes provide a valuable resource for developing complementary molecular markers for PK germplasm discrimination and characterization. However, the present markers require validation across broader and independent PK collections, and complementary nuclear genomic data are needed for a more complete evaluation of genetic diversity and population history.

## Data Availability

The data presented in this study have been deposited in GenBank under accession numbers OR896628–OR896665 and OR492287.

## Supplementary material

Supplementary Figure 1 **(a, b)** The bar plot of the RSCU values for each amino acid, grouped by species. Codons are color-coded, and their corresponding amino acids are labeled below the plot.

Supplementary Figure 2 Number of types of repeat sequences presented in 14 cp genomes. P, F, and R indicate the repeat types.

Supplementary Figure 3 Global comparison of complete genomes of the genus *Polygonatum* coding and non-coding regions are colored in blue and red, respectively.

Supplementary Figure 4 Global comparison of complete genomes of *P. kingianum*. Coding and non-coding regions are colored in blue and red, respectively.

Supplementary Figure 5 A: sliding window analysis of *P. kingianum* cp genome; B: sliding window analysis of *Polygonatum* cp genome. The X-axis represents the window’s midpoint; The Y-axis represents nucleotide diversity values. Window length: 600 bp; step size: 200 bp.

Supplementary Figure 6 Comparisons of the borders of LSC, SSC, and IRa/b regions among the 15 *Polygonatum* cp genomes. The numbers represent the distance between the gene ends, and the border sites, and the numbers below represent the length of the LSC, SSC, and IRa/b regions.

Supplementary Figure 7 The gel electrophoresis and sequencing results of the PCR products. Primer 1 (*ycf2*) was tested. Lane M: marker of DL 2000; 1. *P. kingianum*; 2. *P. sibiricum*; 3. *P. cyrtonema*; 4. *P. odoratum*; 5. *P. hunanense*; 6. *P.cirrhifolium*; 7. *P. franchetii*; 8. *P. verticillatum*; 9. *P. hirtellum.*

Supplementary Figure 8 The gel electrophoresis and sequencing results of the PCR products. Primer 2 (*rps7-trnV*) was tested. Lane M: marker of DL 2000; 1. *P. kingianum*; 2. *P. cyrtonema*; 3. *P. hirtellum*; 4. *P. franchetii*; 5. *P. verticillatum*; 6. *P.cirrhifolium*; 7. *P. sibiricum*; 8. *P. hunanense*; 9. *P. odoratum.*

Supplementary Figure 9 The gel electrophoresis and sequencing results of the PCR products. I: Primer 5 (*atpH-atpF*); II: Primer 6 (*clpP*). Lane M: marker of DL 2000; 1. *P. kingianum*1; 2. *P. kingianum*2; 3. *P. kingianum*3; 4. *P. kingianum*4; 5. *P. kingianum*5; 6. *P. kingianum*6; 7. *P. kingianum*7.

Supplementary Figure 10 BI phylogenetic tree based on 92 cp genomes. The node labels are listed at each node.

Supplementary Figure 11 ML and BI phylogenetic tree based on shared CDS sequence. The node labels are listed at each node.

Supplementary Figure 12 ML phylogenetic trees constructed from six individual hypervariable regions using 92 chloroplast genomes. (a) *ycf1*; (b) *ycf2*; (c) *clpP*; (d) *rps7–trnV*; (e) *atpF–atpH*; and (f) *ycf4–cemA*.

Supplementary Figure 13 ML phylogenetic tree constructed from the concatenated sequences of six hypervariable regions using the same 92 accessions.

Supplementary Table 1 Species information downloaded from NCBI.

Supplementary Table 2 Gene composition in the *Polygonatum* cp genome.

Supplementary Table 3 The base of the codon in the cp genome.

Supplementary Table 4 Distribution of simple sequence repeats (SSRs) among different genomic regions of the chloroplast genomes of Polygonatum species.

Supplementary Table 5 Variations in different regions of the cp genome.

Supplementary Table 6 Dataset design.
