# Supplementary material for: Germplasm evaluation and differentiation in Polygonatum kingianum via chloroplast genome resources
Source: Front Plant Sci. 2026 Oct 15;17:1971675. doi: 10.3389/fpls.2026.1971675 (PMC13716785; doi:10.3389/fpls.2026.1971675)
Supplement: Supplementary file 1 [file Table1.doc]

**Table S1** Species information downloaded by NCBI.

| NO. | Speices | GenBank  accesion | NO. | Speices | GenBank  accesion |
| --- | --- | --- | --- | --- | --- |
| 1 | *P. kingianum* | MN934979.1 | 39 | *P. kingianum* | OR995647.1 |
| 2 | *P. kingianum* | MN962957.1 | 40 | *P. kingianum* | OR995646.1 |
| 3 | *P. kingianum* | MN962958.1 | 41 | *P. kingianum* | OR995645.1 |
| 4 | *P. kingianum* | MN962959.1 | 42 | *P. kingianum* | OR992389.1 |
| 5 | *P. kingianum* | MW373516.1 | 43 | *P. kingianum* | OR992388.1 |
| 6 | *P. kingianum* | MW373517.1 | 44 | *P. kingianum* | OR992387.1 |
| 7 | *P. kingianum* | MW566444.1 | 45 | *P. kingianum* | OR992386.1 |
| 8 | *P. kingianum* | MW566456.1 | 46 | *P. kingianum* | OR995657.1 |
| 9 | *P. kingianum* | MW566464.1 | 47 | *P. kingianum* | OR992385.1 |
| 10 | *P. kingianum* | MW566465.1 | 48 | *P. kingianum* | OR992383.1 |
| 11 | *P. kingianum* | MW566467.1 | 49 | *P. kingianum* | OR992382.1 |
| 12 | *P. kingianum* | MW788495.1 | 50 | *P. kingianum* | OR992381.1 |
| 13 | *P. kingianum* | MZ286312.1 | 51 | *P. kingianum* | OR992380.1 |
| 14 | *P. kingianum* | MZ286313.1 | 52 | *P. kingianum* | OR992379.1 |
| 15 | *P. kingianum* | MZ286314.1 | 53 | *P. kingianum* | OR992378.1 |
| 16 | *P. kingianum* | MZ286315.1 | 54 | *P. kingianum* | OR992377.1 |
| 17 | *P. kingianum* | MZ286316.1 | 55 | *P. kingianum* | OR992376.1 |
| 18 | *P. kingianum* | MZ286317.1 | 56 | *P. kingianum* | OR992375.1 |
| 19 | *P. kingianum* | MZ286318.1 | 57 | *P. kingianum* | OR995656.1 |
| 20 | *P. kingianum* | MZ286319.1 | 58 | *P. kingianum* | OR402097.1 |
| 21 | *P. kingianum* | MZ286320.1 | 59 | *P. kingianum* | OR995658.1 |
| 22 | *P. kingianum* | MZ286321.1 | 60 | *P. kingianum* | OR995655.1 |
| 23 | *P. kingianum* | MZ286322.1 | 61 | *P. kingianum* | OR995654.1 |
| 24 | *P. kingianum* | MZ286323.1 | 62 | *P. kingianum* | OR995653.1 |
| 25 | *P. kingianum* | MZ286324.1 | 63 | *P. kingianum* | OR992384.1 |
| 26 | *P. kingianum* | MZ286325.1 | 64 | *P. kingianum* | OR995652.1 |
| 27 | *P. kingianum* | MZ286326.1 | 65 | *P. kingianum* | OR995651.1 |
| 28 | *P. kingianum* | NC_047406.1 | 66 | *P. kingianum* (*P. ericoideum*) | MW566439.1 |
| 29 | *P. uncinatum* | NC_058568.1 | 67 | *P. kingianum* (*P. huanum*) | MW566440.1 |
| 30 | *P. kingianum* | ON872712.1 | 68 | *P. kingianum* (*P. huanum*) | MW566441.1 |
| 31 | *P. kingianum* | OQ532966.1 | 69 | *P. kingianum* (*P. uncinatum*) | MW566442.1 |
| 32 | *P. kingianum* | OQ532967.1 | 70 | *P. kingianum* (*P. vietnamensis*) | MW566446.1 |
| 33 | *P. kingianum* | OQ532968.1 | 71 | *P. kingianum* (*P. vietnamensis*) | MW566447.1 |
| 34 | *P. kingianum* | OQ532971.1 | 72 | *P. kingianum* (*P. uncinatum*) | MW566448.1 |
| 35 | *P. kingianum* | OR995659.1 | 73 | *P. kingianum* (*P. esquirolii*) | MW566449.1 |
| 36 | *P. kingianum* | OR995650.1 | 74 | *P. kingianum* (*P. esquirolii*) | MW566450.1 |
| 37 | *P. kingianum* | OR995649.1 | 75 | *P. kingianum (P. agglutinatum)* | MW566452.1 |
| 38 | *P. kingianum* | OR995648.1 | 76 | *P. kingianum (P. darrisii)* | MW566453.1 |
| 77 | *P. kingianum* (*P. esquirolii*) | MW566454.1 | 98 | *P. curvistylum* | MZ150837.1 |
| 78 | *P. kingianum* | MW566455.1 | 99 | *P. hookeri* | MZ150845.1 |
| 79 | *P. kingianum* | MW566457.1 | 100 | *P. punctatum* | MZ150862.1 |
| 80 | *P. kingianum (P. cavaleriei)* | MW566458.1 | 101 | *P. filipes* | OL405010.1 |
| 81 | *P. kingianum* (*P. ericoideum*) | MW566459.1 | 102 | *P. hookeri* | OL405012.1 |
| 82 | *P. kingianum (P. cavaleriei)* | MW566462.1 | 103 | *P. humile* | OL405013.1 |
| 83 | *P. kingianum* (*P. darrisii*) | MW566463.1 | 104 | *P. involucratum* | OL405015.1 |
| 84 | *P. kingianum* (*P. ericoideum*) | MW566466.1 | 105 | *P. odoratum* | OL405016.1 |
| 85 | *P. kingianum* (*P. cavaleriei*) | MW566468.1 | 106 | *P. prattii* | OL405017.1 |
| 86 | *P. kingianum* | MZ029091.1 | 107 | *P. zanlanscianense* | OL405020.1 |
| 87 | *P. uncinatum* | MZ150864.1 | 108 | *P. sibiricum* | OL405024.1 |
| 88 | *P. uncinatum* | OL405019.1 | 109 | *P. stenophyllum* | OL405025.1 |
| 89 | *P. stenophyllum* | KX822773.1 | 110 | *P. cyrtonema* | OL436258.1 |
| 90 | *P. cyrtonema* | MW248135.1 | 111 | *P. mengtzense* | OL587680.1 |
| 91 | *P. zanlanscianense* | MW373522.1 | 112 | *P. campanulatum* | ON534060.1 |
| 92 | *P. hunanense* (*P. polyphylla var. grandifolia*) | MW566451.1 | 113 | *P. cyrtonema* | ON534061.1 |
| 93 | *P. hunanense* (*P. polyphylla var. grandifolia*) | MW566460.1 | 114 | *P. franchetii* | ON534063.1 |
| 94 | *P. hunanense (P. polyphylla var. grandifolia)* | MW566461.1 | 115 | *P. sibiricum* | ON534064.1 |
| 95 | *P. cirrhifolium* | MZ029092.1 | 116 | *Maianthemum bifolium* | ON872719.1 |
| 96 | *P. sibiricum* | MZ029093.1 | 117 | *Hetero alternicirrhosum* | MZ150832.1 |
| 97 | *P. cyrtonema* | MZ029094.1 | 118 | *Hetero ogisui* | MZ150833.1 |

**Table S2** Gene composition in the *Polygonatum* cp genome.

| Category for genes | Group of genes | Name of genes |
| --- | --- | --- |
| Self-replication | Ribosomal RNAs | *rrn4.5S*(×2)*, rrn5S*(×2)*, rrn16S*(×2)*, rrn23S*(×2) |
|  | Transfer RNAs | *trnK-UUU, trnQ-UUG, trnS-GCU, trnR-UCU, trnC-GCA, trnD-GUC, trnY-GUA, trnE-UUC, trnT-GGU, trnS-UGA, trnG-GCC, trnfM-CAU, trnS-GGA, trnT-UGU, trnL-UAA, trnF-GAA, trnV-UAC, trnM-CAU, trnW-CCA, trnP-UGG, trnL-UAG, trnG-UCC*, trnH-GUG*(×2)**, trnI-CAU*(×2)**, trnL-CAA*(×2)**, trnV-GAC*(×2)**, trnI-GAU*(×2)**, trnA-UGC*(×2)**, trnR-ACG*(×2)**, trnN-GUU*(×2)*** |
|  | Small subunit of ribosome | *rps11, rps12*(×2)**, rps14, rps15, rps16, rps18, rps19*(×2)**, rps2, rps3, rps4, rps7*(×2)**, rps8* |
|  | Large subunit of ribosome | *rpl14, rpl16, rpl2*(×2)**, rpl20, rpl22, rpl23*(×2)**, rpl32, rpl33, rpl36* |
|  | RNA polymerase | *rpoA, rpoB, rpoC1, rpoC2* |
| Genes for Photosynthesis | Photosystem Ⅰ | *psaA, psaB, psaC, psaI, psaJ* |
|  | Photosystem Ⅱ | *psbA, psbB, psbC, psbD, psbE, psbF, psbI, psbJ, psbK, psbL, psbM, psbN, psbT, psbZ, ycf3*** |
|  | Cytochrome b/f complex | *petA, petB, petD, petG, petL, petN* |
|  | ATP synthase | *atpA, atpB, atpE, atpF, atpH, atpI* |
|  | Rubisco | *rbcL* |
|  | NADH dehydrogenase | *ndhA, ndhB*(×2)**, ndhC, ndhD, ndhE, ndhF, ndhG, ndhH, ndhI, ndhJ, ndhK* |
| Other genes | Maturase | *matK* |
|  | Envelop membrane protein | *cemA* |
|  | acetyl-CoA-carboxylase | *accD* |
|  | c-type cytochrome synthesis | *ccsA* |
|  | Protease | *clpP*** |
| Genes of unknown function | Conserved open reading frames | *ycf1*, ycf2*(×2)**, ycf4* |

Intron-containing genes are marked by asterisks (*)

**Table S3** The base of the codon in the chloroplast genome.

| Sample | T3s | C3s | A3s | G3s | CAI | CBI | Fop | ENc | GC3s |
| --- | --- | --- | --- | --- | --- | --- | --- | --- | --- |
| *P. kingianum*1 | 0.4685 | 0.1717 | 0.4354 | 0.1729 | 0.167 | -0.103 | 0.354 | 49.14 | 0.264 |
| *P. kingianum*2 | 0.4678 | 0.172 | 0.4249 | 0.1738 | 0.167 | -0.099 | 0.354 | 49.22 | 0.267 |
| *P. kingianum*3 | 0.4688 | 0.1684 | 0.4297 | 0.1649 | 0.167 | -0.096 | 0.355 | 48.73 | 0.259 |
| *P. kingianum*4 | 0.4679 | 0.1723 | 0.4247 | 0.1736 | 0.167 | -0.099 | 0.354 | 49.22 | 0.267 |
| *P. kingianum*5 | 0.4681 | 0.1721 | 0.4246 | 0.1738 | 0.167 | -0.099 | 0.354 | 49.22 | 0.267 |
| *P. kingianum*6 | 0.4685 | 0.1717 | 0.4353 | 0.173 | 0.167 | -0.103 | 0.353 | 49.15 | 0.264 |
| *P. Kingianum*7 | 0.4675 | 0.1715 | 0.4269 | 0.171 | 0.167 | -0.098 | 0.354 | 49.13 | 0.265 |
| *P. cyrtonema*1 | 0.4685 | 0.1718 | 0.4318 | 0.1757 | 0.167 | -0.102 | 0.353 | 49.21 | 0.266 |
| *P. cyrtonema*2 | 0.4678 | 0.1722 | 0.4345 | 0.1747 | 0.166 | -0.103 | 0.353 | 49.26 | 0.266 |
| *P. cyrtonema*3 | 0.4686 | 0.1718 | 0.4317 | 0.1757 | 0.167 | -0.102 | 0.353 | 49.21 | 0.266 |
| *P. hirtellum* | 0.4669 | 0.1727 | 0.4251 | 0.1751 | 0.167 | -0.1 | 0.353 | 49.34 | 0.269 |
| *P. franchetii* | 0.4676 | 0.1721 | 0.4353 | 0.173 | 0.166 | -0.103 | 0.353 | 49.19 | 0.265 |
| *P. verticillatum* | 0.4672 | 0.1725 | 0.4257 | 0.1747 | 0.167 | -0.101 | 0.353 | 49.31 | 0.268 |
| *P. cirrhifolium* | 0.4668 | 0.1724 | 0.435 | 0.1738 | 0.167 | -0.103 | 0.353 | 49.23 | 0.266 |

**Table S4** Distribution of simple sequence repeats (SSRs) among different genomic regions of the chloroplast genomes of *Polygonatum* species.

| Sample | CDS | IGS | Intron | tRNA/rRNA | Total SSRs |
| --- | --- | --- | --- | --- | --- |
| *P. kingianum 1* | 23 | 14 | 4 | 5 | 46 |
| *P. kingianum 2* | 20 | 21 | 9 | 4 | 54 |
| *P. kingianum 3* | 22 | 22 | 5 | 4 | 53 |
| *P. kingianum 4* | 19 | 20 | 10 | 3 | 52 |
| *P. kingianum 5* | 18 | 23 | 7 | 4 | 52 |
| *P. kingianum 6* | 21 | 14 | 6 | 6 | 47 |
| *P. kingianum 7* | 27 | 16 | 3 | 6 | 52 |
| *P. cyrtonema 1* | 17 | 19 | 4 | 6 | 46 |
| *P. cyrtonema 2* | 22 | 16 | 4 | 2 | 44 |
| *P. cyrtonema 3* | 17 | 17 | 4 | 6 | 44 |
| *P. hirtellum* | 23 | 11 | 9 | 6 | 49 |
| *P. franchetii* | 26 | 14 | 8 | 7 | 55 |
| *P. verticillatum* | 20 | 13 | 6 | 6 | 45 |
| *P. cirrhifolium* | 31 | 13 | 7 | 6 | 57 |

Abbreviations: CDS, coding sequence; IGS, intergenic spacer

**Table S5** Variations in different regions of the chloroplast genome.

|  | Plastome | LSC region | SSC region | IR regions |
| --- | --- | --- | --- | --- |
| SNPs | 1247 | 842 | 268 | 137 |
| indels | 263 | 200 | 32 | 31 |

**Table S6 Dataset design**

| Analysis | Dataset |
| --- | --- |
| Genome characterization | 39 newly generated cp genomes (Table 1) |
| Interspecific comparison | 39 newly generated cp genomes and three additional reference genomes |
| PK SNP/indel analysis | 118 PK genomes. 88 genomes (No. 1 - 88, Table S1) and 30 (No. 1 - 30, Table 1) |
| Marker development | Nine species. *P. kingianum*, *P. cyrtonema*, *P. hirtellum*, *P. franchetii*, *P. verticillatum*, *P. cirrhifolium,* *P. odoratum*, *P. hunanense*, and *P. sibiricum* |
| Phylogeny | 92 genomes. 39 newly generated genomes (Table 1) and 53 retrieved from NCBI (No. 66–118 in Table S1) |
